# No association between Parkinson disease and autoantibodies against NMDA-type glutamate receptors

**DOI:** 10.1186/s40035-019-0153-0

**Published:** 2019-04-03

**Authors:** Franziska Hopfner, Stefanie H. Müller, Dagmar Steppat, Joanna Miller, Nele Schmidt, Klaus-Peter Wandinger, Frank Leypoldt, Daniela Berg, Andre Franke, Wolfgang Lieb, Lukas Tittmann, Monika Balzer-Geldsetzer, Simon Baudrexel, Richard Dodel, Ruediger Hilker-Roggendorf, Elke Kalbe, Jan Kassubek, Thomas Klockgether, Inga Liepelt-Scarfone, Brit Mollenhauer, Petra Neuser, Kathrin Reetz, Oliver Riedel, Claudia Schulte, Jörg B. Schulz, Annika Spottke, Alexander Storch, Claudia Trenkwalder, Hans-Ulrich Wittchen, Karsten Witt, Ullrich Wüllner, Günther Deuschl, Gregor Kuhlenbäumer

**Affiliations:** 1 Department of Neurology, University Hospital Schleswig Holstein, Arnold-Heller Str. 3, 24105 Kiel, Germany; 2 Center of Blood Transfusion, University Hospital Schleswig Holstein, Kiel & Lübeck, Germany; 3 Department of Clinical Chemistry, University Hospital Schleswig-Holstein, Kiel, Germany; 4 Institute of Clinical Molecular Biology, University Hospital Schleswig-Holstein, Kiel, Germany; 5 Institute of Epidemiology, University Hospital Schleswig-Holstein, Kiel, Germany; 6 Chair of Geriatric Medicine, University Hospital Essen, Essen, Germany; 7 Department of Neurology, Philipps-University Marburg, Marburg, Germany; 8 Department of Neurology, Goethe University, Frankfurt/Main, Germany; 9 Brain Imaging Center, Goethe University, Frankfurt/Main, Germany; 10 Department of Medical Psychology | Neuropsychology and Gender Studies & Center for Neuropsychological Diagnostics and Intervention (CeNDI), University Hospital Cologne, Cologne, Germany; 11 Psychological Gerontology, Institute of Gerontology, University of Vechta, Vechta, Germany; 12 Department of Neurology, University of Ulm, Ulm, Germany; 13 Department of Neurology, University of Bonn, and German Center for Neurodegenerative Diseases (DZNE), Bonn, Germany; 14 Center for Neurology, Hertie Institute for Clinical Brain Research, Department of Neurodegenerative Diseases, University of Tübingen, Tübingen, Germany; 15 DZNE, German Center for Neurodegenerative Diseases, Tübingen, Germany; 16 Paracelsus-Elena-Klinik, Kassel, Germany; 17 Institute of Neuropathology and Department of Neurosurgery, University Medical Center Göttingen, Göttingen, Germany; 18 Coordinating Centre for Clinical Trials, Philipps-University, Marburg, Germany; 19 Department of Neurology, RWTH Aachen University, Aachen, Germany; 20 JARA-BRAIN Institute of Molecular Neuroscience and Neuroimaging, Forschungszentrum Jülich GmbH and RWTH Aachen University, Aachen, Germany; 21 Leibniz Institute for Prevention Research and Epidemiology – BIPS, Achterstrasse 30, 28359 Bremen, Germany; 22 Department of Neurology, Technische Universität Dresden, 01307 Dresden, Germany; 23 German Center for Neurodegenerative Diseases (DZNE), Research Site Rostock, 18147 Rostock, Germany; 24 Department of Neurology, University of Rostock, 18147 Rostock, Germany; 25 Technische Universtität Dresden, Institute of Clinical Psychology and Psychotherapy, Chemnitzer Str. 46, 01187 Dresden, Germany; 26 Technische Universtität Dresden, Center for Epidemiological and Longitudinal Studies (CELOS), Chemnitzer Str. 46, 01187 Dresden, Germany; 27 Department of Psychiatry and Psychotherapy, Ludwig-Maximilians-Universität München, Nußbaumstraße 7, 80336 München, Germany; 28 Department of Neurology, European Medical School, University Oldenburg, Oldenburg, Germany; 29 Research Center Neurosensory Science, Carl von Ossietzky University Oldenburg, Oldenburg, Germany

**Keywords:** NMDA antibody, NMDA IgA/IgM antibodies, Parkinson disease, Cognitive impairment

## Abstract

**Background:**

IgG-class autoantibodies to N-Methyl-D-Aspartate (NMDA)-type glutamate receptors define a novel entity of autoimmune encephalitis. Studies examining the prevalence of NMDA IgA/IgM antibodies in patients with Parkinson disease with/without dementia produced conflicting results. We measured NMDA antibodies in a large, well phenotyped sample of Parkinson patients without and with cognitive impairment (*n* = 296) and controls (*n* = 295) free of neuropsychiatric disease. Detailed phenotyping and large numbers allowed statistically meaningful correlation of antibody status with diagnostic subgroups as well as quantitative indicators of disease severity and cognitive impairment.

**Methods:**

NMDA antibodies were analysed in the serum of patients and controls using well established validated assays. We used anti-NMDA antibody positivity as the main independent variable and correlated it with disease status and phenotypic characteristics.

**Results:**

The frequency of NMDA IgA/IgM antibodies was lower in Parkinson patients (13%) than in controls (22%) and higher than in previous studies in both groups. NMDA IgA/IgM antibodies were neither significantly associated with diagnostic subclasses of Parkinson disease according to cognitive impairment, nor with quantitative indicators of disease severity and cognitive impairment. A positive NMDA antibody status was positively correlated with age in controls but not in Parkinson patients.

**Conclusion:**

It is unlikely albeit not impossible that NMDA antibodies play a significant role in the pathogenesis or progression of Parkinson disease e.g. to Parkinson disease with dementia, while NMDA IgG antibodies define a separate disease of its own.

**Electronic supplementary material:**

The online version of this article (10.1186/s40035-019-0153-0) contains supplementary material, which is available to authorized users.

## Background

Parkinson disease (PD) is a neurodegenerative disease. Important components of the pathomechanism are protein aggregation and lysosomal as well as mitochondrial dysfunction [1]. Inflammation has been increasingly recognized as an additional component of the pathomechanism of PD [2]. Encephalitis mediated by autoantibodies against NMDA-type glutamate receptors (NMDAab) is an important cause of autoimmune encephalitis [3]. These findings prompted the question whether NMDAab contribute to the inflammatory component of PD. Two previous studies have examined the prevalence of NMDAab in the serum of PD patients. One performed in a large sample of PD patients (*n* = 258) and controls (*n* = 1730) did not find an association of NMDAab with PD [4]. However, in this study detailed phenotypic characteristics were not reported and the study was criticised for using unselected blood-donors as controls [4]. The other study included 74 PD patients, 25 of whom were diagnosed as PD with dementia (PD-D) and 47 healthy controls [5]. This study did not find an association between PD per se and antibody positivity but reported a significantly higher proportion of antibody positive cases in PD patients with dementia compared to PD without dementia [5].

Landscape is a prospective study using detailed phenotyping to characterize the natural course of PD, especially with respect to dementia. Popgen is a population based study of health in northern Germany. The present study uses serum samples of Landscape and popgen Biobank [6] as well as samples collected at the Department of Neurology, Kiel University to address three questions: (I) Are IgA/IgM NMDAab associated with PD?; (II) Does the frequency of NMDAab of the IgA/IgM classes differ between PD patients without cognitive impairment and those with cognitive impairment?; (III) Is IgA/IgM NMDAab antibody status correlated with differences in cognitive test results?

## Participants and methods

### Participants

All participants gave written informed consent. Ethics committee approval was obtained for all studies involved (Department of Neurology of Kiel University, Landscape, popgen). Objectives, recruitment and phenotyping procedures in popgen and Landscape have been described in detail elsewhere [6, 7]. We used the following general metrics: sex, age at examination, age at PD onset, PD duration and Unified Parkinson Disease Rating Scale part III (motor part). Landscape involves a large number of cognitive tests. For this study we analysed the results of the following tests measuring different cognitive domains: Mini-Mental-State-Examination (MMSE) [8], Parkinson Neuropsychometric Dementia Assessment (PANDA) a test especially developed to assess cognitive deficits in PD [9], the backwards digit span (working memory, Wechsler Memory Scale revised) and CERAD word list learning and recall (immediate and delayed verbal memory), the Stroop colour word, colour line and interference test, the CERAD plus trail making test A and B (executive function) [10], the modified Card Sorting Test examining the ability to display flexibility in the face of changing schedules of reinforcement and the Performance Evaluation System for Seniors (LPS 50+) subtest 9 [11], a German test battery with subtest 9 assessing visuo-spatial skills.

In the Landscape study PD with minimal cognitive impairment (PD-MCI) was defined according to established MCI criteria. These included in short (1) cognitive dysfunction reported by the patient, (2) no significant impairment in daily living and (3) at least one score ≥ 1.5 standard deviations (SD) below normative values in at least one of the tests used for diagnosis by Kalbe et al. [12]. PD-D was diagnosed using the criteria for possible and probable PD-D by Emre et al. [13] including (1) cognitive dysfunction reported by the patient or caregiver, (2) significant impairment in daily living and (3) at least two scores ≥1.5 standard deviations (SD) below normative values in two of five different cognitive domains. Features suggesting other conditions or diseases as cause of mental impairment were exclusion criteria for the diagnosis PD-D. This study analysed 296 PD patients (93 from the Departmentof Neurology Kiel University, 203 from the Landscape study) and 295 controls (49 from the Department of Neurology Kiel University, 246 from the popgen study).

Serum samples and phenotypes of Landscape patients were obtained from the central repository at Marburg University. Popgen controls were nearly perfectly matched with respect to sex and age to the patients and serum samples obtained from the popgen biobank at Kiel University [6]. PD patients and sex as well as approximately age matched controls from the Department of Neurology, Kiel University were prospectively collected exclusively by FH. FH assessed the following phenotypes in PD patients: age, sex, UPDRS III, dementia (no, yes), mild cognitive impairment (MCI, no, yes) by neuropsychiatric examination and via consultation of the clinical records. FH examined the Kiel University controls rendering dementia and severe mood disorders unlikely. All controls were free of self-reported neuropsychiatric disease. All popgen controls underwent a standardized general physical examination by the study physicians and completed questionnaires including screening questions for mood disorders which revealed no evidence for a neuropsychiatric disease or dementia. However, formal neuropsychological testing for dementia was not performed in either control group and mood disorders were not assessed in patients as well as Kiel controls.

### Serological analyses

Serum samples of all participants were processed according to previously published, validated procedures also used in both previous studies at Euroimmun, Lübeck, Germany [4, 5]. Serum was tested at a starting dilution of 1:10 on fixed transfected HEK-cells using FITC labelled goat-anti-human Ig detecting all isoforms. Positive samples were further assessed using Fc-specific anti-human IgG, IgA or IgM and performing serial dilutions according to manufacturer’s instructions (Euroimmun). End-point titres were assessed by researchers blinded to clinical data. In addition to NMDAab IgG, IgA and IgM, a number of additional autoantibodies were assessed. None of them was prevalent enough to perform a meaningful statistical analysis (data not shown).

### Statistical analyses

All statistical analyses were performed using RStudio (version 1.0.136). Detailed data on NMDAab titres are found in Additional file 1: Table S1. For comparability with previous studies we regarded all samples with any titre of NMDAab as positive. However, we also performed the analyses regarding only titres > 1:32 as positive because the value of low NMDAab titres is a matter of debate. Both definitions of NMDAab positivity revealed essentially the same results, meaning that *p*-values changed but none of the insignificant differences between groups became significant or vice versa (data not shown).

For comparison of categorical values between groups, we used the Chi-square test (chi2). Age at examination, age at PD onset and PD duration were compared using Student’s t-test (t) for two groups and analysis of variance (ANOVA) for more than two groups. Non-normally distributed interval-scaled or ordinal data were compared using the Mann-Whitney-U test (MWU) for two groups and the Kruskal-Wallis test (KW) for more than two groups. The quantitative relationship between age (exposure) and antibody status (outcome) was assessed using logistic regression (LR) in order to obtain odds-ratios.

## Results

It is now well recognized that NMDAab receptor encephalitis is a separate etiologic entity caused only by specific IgG but not by IgA or IgM NMDAab [3]. Therefore we excluded IgG NMDAab from the analysis. However, since only one PD and two controls had positive IgG NMDAab, including or excluding them had only extremely minor impact on the results which was far from changing any statistically insignificant differences to significant ones or vice versa (data not shown). In the following parts we will therefore refer to NMDAab of the IgA and IgM subclasses in our sample as NMDAab.

Table 1 compares different demographic characteristics., The UPDRS III score, results of cognitive tests and the frequency of NMDAab between PD patients (*n* = 296) and controls (*n* = 295) as well as following diagnostic subgroups: PD without cognitive impairment (PD-WOC), PD with MCI (PD-MCI), PD with dementia (PD-D) had been compared. Case-control matching resulted in nearly perfect sex matching and minimal albeit significant age difference (~ 2 years) between PD patients and controls. Since increasing age has repeatedly been associated with an increase in NMDAab antibody frequency we examined the influence of age, using a logistic regression model with NMADab positivity as outcome variable [4, 14]. NMDAab positivity was related to increasing age in controls (*p* = 0.009, OR = 1.07, 95%CI: 1.02–1.13). However, age had no significant influence on NMDAab positivity in PD patients (*p* = 0.599, OR = 0.987, 95%CI: 0.939–1.039). Therefore, we did not use regression models with age as covariate for all analyses performed within PD patient subgroups only. Among PD patients, the age at examination, the age at PD onset, the duration of PD at the time of examination and the UPDRS III score increased in the order PD-WOC, PD-MCI and PD-D (Table 1). All measures of cognitive performance indicated an increasing cognitive deficit from PD-WOC to PD-MCI and PD-D (Table 1). NMDAab were more frequent in controls (22%) than in PD patients (13%, *p* = 0.003) as well as in controls compared to each of the three PD patient diagnostic subgroups (Table 1). NMDAab frequency did not differ significantly between the diagnostic subgroups (Table 1, *p* = 0.885) and also not comparing PD-WOC against all PD with cognitive impairment (PD-MCI + PD-D, *p* = 0.662) nor comparing PD-WOC against PD-D (*p* = 0.937). Table 2 shows the metrics and *p*-values of quantitative tests in NMDAab negative versus NMDAab positive PD patients. Age at examination, age at PD onset and PD duration as well as the cognitive test results did not differ significantly between NMDAab negative and NMDAab positive PD patients (Table 2). Finally, Additional file 1: Table S1 provides frequencies and titres of NMDAab sub-classes (IgA, IgM) in PD patients, control subjects and the three diagnostic subgroups.

**Table 1:** Study metrics for PD and controls as well and diagnostic subgroups

Diagnostic group	Value	Test	Comparison	*P*-value	PD	PD-WOC	PD-MCI	PD-D	All controls
Male	n (%)	chi2	all PD vs. Controls	0.9747	186 (63)	89 (59)	77 (68)	20 (63)	185 (63)
Mean Sample age in years	mean/sd	t.test	all PD vs. Controls	0.0007	68 ± 7	66 ± 7	69 ± 5	73 ± 6	66 ± 6
Age PD onset	mean/sd	ANOVA	PD-WOC, PD-MCI, PD-D	0.0103	60 ± 9	59 ± 8	61 ± 8	63 ± 10	na
PD duration	mean/sd	ANOVA	PD-WOC, PD-MCI, PD-D	0.0930	8.3 ± 5.9	7.6 ± 5.2	8.7 ± 5.9	9.9 ± 7.7	na
UPDRS III	median/mad	KW	PD-WOC, PD-MCI, PD-D	3.06E-06	21 ± 11.9	18 ± 10.4	22 ± 11.9	31 ± 14.1	na
MMSE	median/mad	KW	PD-WOC, PD-MCI, PD-D	9.34E-12	29 ± 1.5	29 ± 1.5	28 ± 1.5	25 ± 3.0	na
PANDA	median/mad	KW	PD-WOC, PD-MCI, PD-D	3.40E-12	23 ± 5.9	26 ± 4.5	22 ± 4.5	13 ± 7.4	na
Wechsler Memory Scale digit span reverse, number of correct numerical series	median/mad	KW	PD-WOC, PD-MCI, PD-D	3.21E-07	6 ± 1.5	6 ± 1.5	5 ± 1.5	4 ± 3.0	na
Modified Card Sorting Test, Categories completed	median/mad	KW	PD-WOC, PD-MCI, PD-D	4.2E-11	4 ± 1.5	5 ± 1.5	3 ± 1.5	2 ± 1.5	na
Stroop Test reading colours (s)	median/mad	KW	PD-WOC, PD-MCI, PD-D	6.67E-08	36 ± 6.7	32 ± 5.9	38 ± 7.4	49 ± 14.8	na
Stroop Test naming colours (s)	median/mad	KW	PD-WOC, PD-MCI, PD-D	1.11E-06	53 ± 11.9	50 ± 8.9	55 ± 14.1	62 ± 8.9	na
Stroop Test interference (s)	median/mad	KW	PD-WOC, PD-MCI, PD-D	9.71E-09	101 ± 23.7	90 ± 19.3	105 ± 28.9	161 ± 68.9	na
CERAD Trail Making Test A, time to complete (s)	median/mad	KW	PD-WOC, PD-MCI, PD-D	2.31E-13	45 ± 17.8	38 ± 10.4	51 ± 17.8	96 ± 46.7	na
CERAD Trail Making Test B, time to complete (s)	median/mad	KW	PD-WOC, PD-MCI, PD-D	2.31E-13	120 ± 63.8	92 ± 36.3	141.0 ± 67.5	275 ± 37.1	na
CERAD word list memory test, sum score of 3 trails, immediate recall (range 0–30)	median/mad	KW	PD-WOC, PD-MCI, PD-D	4.75E-11	19 ± 4.5	22 ± 4.5	19 ± 4.5	14 ± 5.9	na
CERAD word list, delayed free recall (range 0–10)	median/mad	KW	PD-WOC, PD-MCI, PD-D	5.66E-11	7 ± 3.0	8 ± 1.5	6 ± 3.0	4 ± 1.5	na
Performance Evaluation System for Seniors (LPS 50+) subtest spatial abilities, sum of correct items	median/mad	KW	PD-WOC, PD-MCI, PD-D	3.52E-13	18 ± 7.4	21 ± 4.5	15 ± 7.4	5 ± 4.4	na
NMDA positive	n (%)	chi2	all PD vs. Controls	0.0032	38(13)/258(87)	na	na	na	65(22)/230(78)
NMDA positive	n (%)	chi2	PD-WOC, PD-MCI, PD-D	0.8854	na	18(12)/132(88)	16(14)/98(86)	4(13)/28(87)	na
NMDA positive	n (%)	chi2	PD-WOC vs. (PD-MCI + PD-D)	0.6622	na	18(12)/132(88)	20(14)/126(86)		na
NMDA positive	n (%)	chi2	PD-WOC vs. PD-D	0.9372	na	18(12)/132(88)	na	4(13)/28(87)	na

**Table 2:** Comparison of demographic, clinical and cognitive parameters between NMDAab negative and NMDAab positive PD patients

Metric between nmda negative and nmda positive PD patients	Value	Test	*P*-value
Sample age	mean/sd	t-test	0.655
Age PD onset	mean/sd	t-test	0.316
PD duration	mean/sd	t-test	0.401
UPDRS III	median/mad	MWU	0.723
MMSE	median/mad	MWU	0.808
PANDA	median/mad	MWU	0.174
Wechsler Memory Scale digit span reverse, number of correct numerical series	median/mad	MWU	0.259
Modified Card Sorting Test, Categories completed	median/mad	MWU	0.325
Stroop Test reading colours (s)	median/mad	MWU	0.190
Stroop Test naming colours (s)	median/mad	MWU	0.390
Stroop Test interference (s)	median/mad	MWU	0.589
CERAD Trail Making Test A, time to complete (s)	median/mad	MWU	0.075
CERAD Trail Making Test B, time to complete (s)	median/mad	MWU	0.167
CERAD word list memory test, sum score of 3 trails, immediate recall (range 0–30)	median/mad	MWU	0.163
CERAD word list, delayed free recall (range 0–10)	median/mad	MWU	0.308
Performance Evaluation System for Seniors (LPS 50+) subtest spatial abilities, sum of correct items	median/mad	MWU	0.070

## Discussion

In this study, NMDAab are not associated with PD or with progression to PD-MCI or PD-D. In contrast, NMDAab are statistically significantly more common in control sera (22%) than in PD patient sera (13% in all PD patients, 12% in PD patients without cognitive impairment). The first of two sizeable previous studies found NMDAab in 2% of PD patients without dementia [5] and the second one in 8.1% [4] of all included PD patients while 4.3% [5] and 8.5% of controls exhibited NMDAab, respectively. These numbers show that the variability in NMDAab frequency between studies is very high. The control subjects in this study were free of self-reported neurological disease. Therefore neurological disease does not account for the high frequency of NMDAab in controls. Age and sex distribution between PD and controls was very similar with a small but statistically significant age difference between groups (Table 1). The high variability of NMDAab frequencies between studies (PD: 2 to 13%, Control: 4.3 to 22%) remains unexplained. All three available studies agree that the frequency of NMDAab in PD patients without dementia is not elevated. However, the study by Doss et al. reports a significantly higher prevalence of NMDAab in PD-D than in PD, which is not found in this study. The PD patients included in this study stem from two sources: the Landscape study focussing on the development of dementia during progression of PD and patients collected at the Department of Neurology of Kiel University. The UPDRS III score at the time of serum sampling and a diagnostic sub-classification into PD-WOC, PD-MCI and PD-D was available for all PD patients. The UPDRS III score showed no significant difference between NMDAab negative and positive PD indicating that the motor symptoms in NMDAab positive PD patients were not more severe than the symptoms of NMDAab negative PD patients. NMDAab were also not significantly more common in PD-D than in PD-WOC or PD-MCI or in PD-WOC versus all PD with cognitive impairment (PD-MCI + PD-D). This finding is in stark contrast to the study by Doss et al. [5] who found that NMDAab were 10-times more common (20% vs. 2%) in PD-D compared to PD without dementia. It should be taken into account that 2% of PD patients without dementia corresponded 1 of 49 samples and 20% of PD-D patients corresponded to 5 out of 25 samples in the study by Doss et al. [5]. The corresponding numbers in our study are also small with 18 of 150 NMDAab positive PD patients without cognitive impairment and 20 of 150 NMDAab positive PD patients with any cognitive impairment, albeit much larger than in the previous study. A plethora of cognitive test results was available for the Landscape study. None of cognitive test results differed significantly between NMDAab positive and negative PD patients. Only the subtest “visuospatial functioning” of the Performance Evaluation systems for Seniors (LPS 50+) showed a suggestive difference (*p* = 0.070). However, regarding this and some of the other tests, PD patients with NMDAab in our sample performed slightly better than patients without NMDAab. We conclude that cognitive impairment in PD including multiple markers of cognitive performance is not correlated with NMDAab in our study. We have not formally tested controls for cognitive impairment which might influence the comparisons between cases and controls. Therefore, we think that the most important added value compared to previous studies is the within-case analysis which is not influenced by control selection and did not reveal an association between antibody status and cognitive test results. Strengths of our study are the large sample size of 296 PD patients and 295 controls, the detailed, especially cognitive phenotyping of PD patients from the Landscape study and the high proportion of population based controls from the popgen study as well as the absence of self-reported neurological disease in all controls. Age and sex matching and an analysis of the influence of age on NMDAab status was undertaken to prevent age/sex effects. Despite these precautions we found an unusually high number of NMDAab carriers among controls which might be related to the lack of controls in the Landscape study necessitating the use of popgen controls collected in a different setting. This represents a major weakness of the study.

## Conclusions

We conclude that we did not find a significantly increased frequency of NMDAab in PD patients with cognitive impairment compared to those without it and that NMDAab status is not correlated with the performance in any of the cognitive tests employed in the Landscape study. However, these findings do still not completely rule out a role for NMDAab in PD.

## Additional file

Additional file 1: **Table S1.** Antibody subclasses, number of NMDAab positive samples and titres in PD patients, controls and PD diagnostic subgroups. (DOCX 14 kb)

## Abbreviations

CERAD: Consortium to establish a registry for Alzheimer’s disease
MCI: Mild cognitive impairment
MMSE: Mini-Mental-State-Examination
NMDAab: Autoantibodies against NMDA-type glutamate receptor
PANDA: Parkinson Neuropsychometric Dementia Assessment
PD: Parkinson disease
PD-D: PD with dementia
PD-MCI: PD with MCI
PD-WOC: PD without cognitive impairment
UPDRS: Unified Parkinson’s Disease Rating Scale
