# Supplementary material for: No association between Parkinson disease and autoantibodies against NMDA-type glutamate receptors
Source: Transl Neurodegener. 2019 Apr 3;8:11. doi: 10.1186/s40035-019-0153-0 (PMC6446289; doi:10.1186/s40035-019-0153-0)
Supplement: Supplementary file 1 — Table S1. Antibody subclasses, number of NMDAab positive samples and titres in PD patients, controls and PD diagnostic subgroups. (DOCX 14 kb) [file 40035_2019_153_MOESM1_ESM.docx]

Additional file 1: Table S1: Antibody subclasses, number of NMDAab positive samples and titres in PD patients, controls and PD diagnostic subgroups.

|  |  | **PD**  **(n = 296)** | **PD-WOC**  **(n = 150)** | **PD-MCI**  **(n = 114)** | **PD-D**  **(n = 32)** | **Control**  **(n = 295)** |
| --- | --- | --- | --- | --- | --- | --- |
|  |  | NMDAab positive samples per group (n) | | | | |
| **IgA** | **total** | 23 | 11 | 8 | 4 | 26 |
|  | **1:10** | 6 | 5 | 1 | 0 | 8 |
|  | **1:32** | 2 | 1 | 1 | 0 | 4 |
|  | **1:100** | 4 | 1 | 0 | 3 | 7 |
|  | **1:320** | 8 | 2 | 6 | 0 | 5 |
|  | **1:1000** | 3 | 2 | 0 | 1 | 2 |
| **IgM** | **total** | 26 | 13 | 12 | 1 | 56 |
|  | **1:10** | 6 | 3 | 3 | 0 | 10 |
|  | **1:32** | 8 | 4 | 4 | 0 | 7 |
|  | **1:100** | 7 | 2 | 4 | 1 | 15 |
|  | **1:320** | 5 | 4 | 1 | 0 | 11 |
|  | **1:1000** | 0 | 0 | 0 | 0 | 7 |
|  | **1:3200** | 0 | 0 | 0 | 0 | 6 |
